# Non-graphitized carbon/Cu_2_O/Cu^0^ nanohybrids with improved stability and enhanced photocatalytic H_2_ production

**DOI:** 10.1038/s41598-023-41211-4

**Published:** 2023-08-26

**Authors:** Areti Zindrou, Loukas Belles, Maria Solakidou, Nikos Boukos, Yiannis Deligiannakis

**Affiliations:** 1 Laboratory of Physical Chemistry of Materials & Environment, Department of Physics, University of Ioannina, Ioannina, Greece; 2 Institute of Nanoscience and Nanotechnology (INN), NCSR Demokritos, 15310 Athens, Greece

**Keywords:** Energy science and technology, Materials science, Nanoscience and technology

## Abstract

Cu_2_O is a highly potent photocatalyst, however photocorrosion stands as a key obstacle for its stability in photocatalytic technologies. Herein, we show that nanohybrids of Cu_2_O/Cu^0^ nanoparticles interfaced with non-graphitized carbon (nGC) constitute a novel synthesis route towards stable Cu-photocatalysts with minimized photocorrosion. Using a Flame Spray Pyrolysis (FSP) process that allows synthesis of anoxic-Cu phases, we have developed in one-step a library of Cu_2_O/Cu^0^ nanocatalysts interfaced with nGC, optimized for enhanced photocatalytic H_2_ production from H_2_O. Co-optimization of the nGC and the Cu_2_O/Cu^0^ ratio is shown to be a key strategy for high H_2_ production, > 4700 μmoles g^−1^ h^−1^ plus enhanced stability against photocorrosion, and onset potential of 0.234 V vs. RHE. After 4 repetitive reuses the catalyst is shown to lose less than 5% of its photocatalytic efficiency, while photocorrosion was < 6%. In contrast, interfacing of Cu_2_O/Cu^0^ with graphitized-C is not as efficient. Raman, FT-IR and TGA data are analyzed to explain the undelaying structural functional mechanisms where the tight interfacing of nGC with the Cu_2_O/Cu^0^ nanophases is the preferred configuration. The present findings can be useful for wider technological goals that demand low-cost engineering, high stability Cu-nanodevices, prepared with industrially scalable process.

## Introduction

Controlled synthesis of metal oxide nanoparticles (NPs) with targeted characteristics allows their applications in diverse fields including biotechnology^1^, optoelectronics^2^ and energy conversion^3^. Tunable nanophases and their heterojunctions is a highly pursued strategy for performance optimization^4^. Ideally, low-cost, non-noble metal oxides are envisioned as catalysts in energy- and chemical- technologies^5^. Copper nanocatalysts are particularly interesting, since, in addition to low-cost and high-abundance, they may attain unique photocatalytic properties. Specifically, cupric oxide (CuO) semiconductor with an energy gap in the range E_g_ = 1.3–1.5 eV^6^ can absorb a substantial part of the solar- light spectrum, finds many applications in gas sensing^7^, catalysis^8^ and as anode- material for lithium ion batteries^9^. Cuprous oxide, (Cu_2_O), as a direct band-gap, *p*-type, semiconductor with E_g_ = 2–2.2 eV^10^, is a promising Cu^1+^-oxide with potential applications in catalysis^11^, electronics^12^ and gas sensors^13^ among others. Within the context of photocatalysis, the conduction band of Cu_2_O lies at E_CB_ = − 1eV vs NHE (pH = 0)^14^ thus is negative enough, and can potentially reduce CO_2_ to useful liquid chemical fuels, i.e., formic acid (HCOOH), formaldehyde (HCHO), methanol (CH_3_OH) to name a few^15^.

Despite these appealing properties, it is well documented that under photocatalytic conditions, Cu NPs lack long-term stability due to photocorrosion, lattice destabilization and deterioration of photocatalytic performance^10^. Photocorrosion can be due to reduction of Cu_2_O to Cu^0^, however it is the oxidation of Cu_2_O to CuO^14^, that is considered as primary source of lattice deterioration i.e. via formation of Cu^2+^-ions^16^. To address photocorrosion, several works discuss the controlled synthesis of stable Cu NPs^17–19^ based on strategies e.g. such as their incorporation in core–shell structures using other oxides^20,21^, or interfacing the Cu-catalysts in the so-called Z-schemes^11,22^, that can inhibit photocorrosion, via sequestration of strongly oxidizing or reducing holes/electrons^23^ respectively. To minimize oxidation of Cu_2_O by the photoinduced holes, the use of appropriate hole scavengers with suitable oxidation potentials, can improve the overall stability of the catalytic system, without addition of metal cocatalyst^24^.

In this context, graphitic carbon layers have been shown to offer protection via collection of the photoinduced electrons. Sun et al*.*^25^ developed an heterostructure of Cu_2_O supported on a 3D g-C_3_N_4_ where at the interface of (111) facet with g-C_3_N_4_, additional DOS were created. In this work^25^ photoexcited electrons were transferred from g-C_3_N_4_ to Cu_2_O avoiding photocorrosion of Cu_2_O and ensuring enhanced photocatalytic activity and high reusability, up to 5 times. In another work, An et al*.*^26^ studied the effect of rGO interfaced with different facets of Cu_2_O. Their composite material showed minimal Cu-atom leaching of ~ 3% after 3h of irradiation and smaller ohmic resistance, indicating better charge transfer comparing to their reference Cu_2_O^26^. On the other hand, non-graphitized carbon can also—in principle—protect from photocorrosion, however although useful its use has not been reported so far. The key-hypothesis is that non-graphitized carbon, at low concentrations, does not intervene to the availability of the photoinduced electrons, at the same time offering a reduction microenvironment at the Cu_2_O, that could minimizes the tendency to be oxidized to CuO. Herein this hypothesis has been investigated as an alternative, novel concept, towards synthesis of stable Cu_2_O nanocatalysts.

Regarding the synthesis of Cu-based nanomaterials, existing methods can be classified into wet chemical methods^27^, microwave assisted methods^28^, sonochemical^29^ and thermal^30^. However, all the above-mentioned techniques, lack scalability at an industrial level e.g., due to the requirement of multiple-steps and/or limited rates of production yields. In this context, Flame Spray Pyrolysis (FSP) process^31^ can be of outmost importance i.e. as a well-established and versatile technology, imminently suited for continuous industrial-scale (kg h^−1^) production of nanomaterials with controlled characteristics. Until today, synthesis of CuO NPs by FSP is well documented. Waser et al*.*^32^*,* have used FSP to synthesize CuO NPs for lithium-ion battery materials^33^. Zhu et al.^34^ reported the successfull synthesis of CuO with a fraction of Cu_2_O-phase via FSP. This CuO-{Cu_2_O} system exhibited notable photocurrent response, attributed by the authors to visible-light absorption and the effective separation of the photogenerated pairs by the CuO–Cu_2_O heterojunction^34^.

So far, synthesis of reduced Cu-nanophases i.e. pure Cu_2_O or Cu_2_O/Cu^0^ NPs by FSP, has not been reported, and this can be attributed to preferable formation of stable CuO in oxygen-rich flames typically used in classical FSP^35^. Grass et al.^36,37^ have shown that controlled Oxygen-content in the combustion FSP atmosphere, enables the synthesis of pure metallic, Co and Bi, nanoparticles. By using a reducing FSP set up Athanassiou et al.^38^ were able to produce metallic Cu^0^ NPs covered by thin (~ 1 nm, 2–3 layers) carbon layer that exhibited excellent air- and acid- stability^41^ however these were not evaluated nor optimized as photocatalysts. The above-mentioned FSP set up was based on an FSP reactor enclosed in a glove-box that involved N_2_-gas as reducing agent. Recently, we have presented an alternative FSP configuration^16,39^ that allows synthesis of reduced metal oxides. This Anoxic-FSP process (A-FSP) has been successfully used to produce highly-photoactive ZrO_2−x_^39^, or high purity Cu_2_O nanoparticles^16^. The process is based on incorporation of methane (CH_4_) as dispersion gas and nitrogen (N_2_) as sheath gas, in combination with an oxygen-lean atmosphere, allowing the in-situ formation of suboxic metal oxides or pure metallic phases.

Herein, we have used the A-FSP process for the synthesis of Cu_2_O/Cu^0^ NPs with controlled amounts of non-graphitized carbon (nGC). In this way, we have produced a library of {nGC/Cu_2_O/Cu^0^} nanohybrids, that contained a range of nGC, 0 to 8% vs. Cu-oxide. For comparison, {GC/Cu_2_O/Cu^0^} nanohybrids codenamed #Cu-G i.e., with graphitized-carbon have also been engineered. Evaluation of photocatalytic H_2_ production from H_2_O, reveals that, optimized {nGC/Cu_2_O/Cu^0^} nanohybrids achieve remarkable H_2_ production rates i.e., 4788umoles H_2_ per gram per hour, that is superior vs. the {GC/Cu_2_O/Cu^0^} nanohybrids. Electrochemical analysis reveals that the {nGC/Cu_2_O/Cu^0^} nanohybrids benefit from inhibited photocorrosion, in accordance with the reusability of the photocatalysts, for up to four times. These data are discussed within the context of the beneficial role of tight interfacing of nGC with the Cu_2_O/Cu^0^ achieved by the A-FSP process in one step.

## Results

### Synthesis of nGC/Cu_2_O/Cu^0^ heterojunctions by anoxic-flame spray pyrolysis

**Figure 1:**
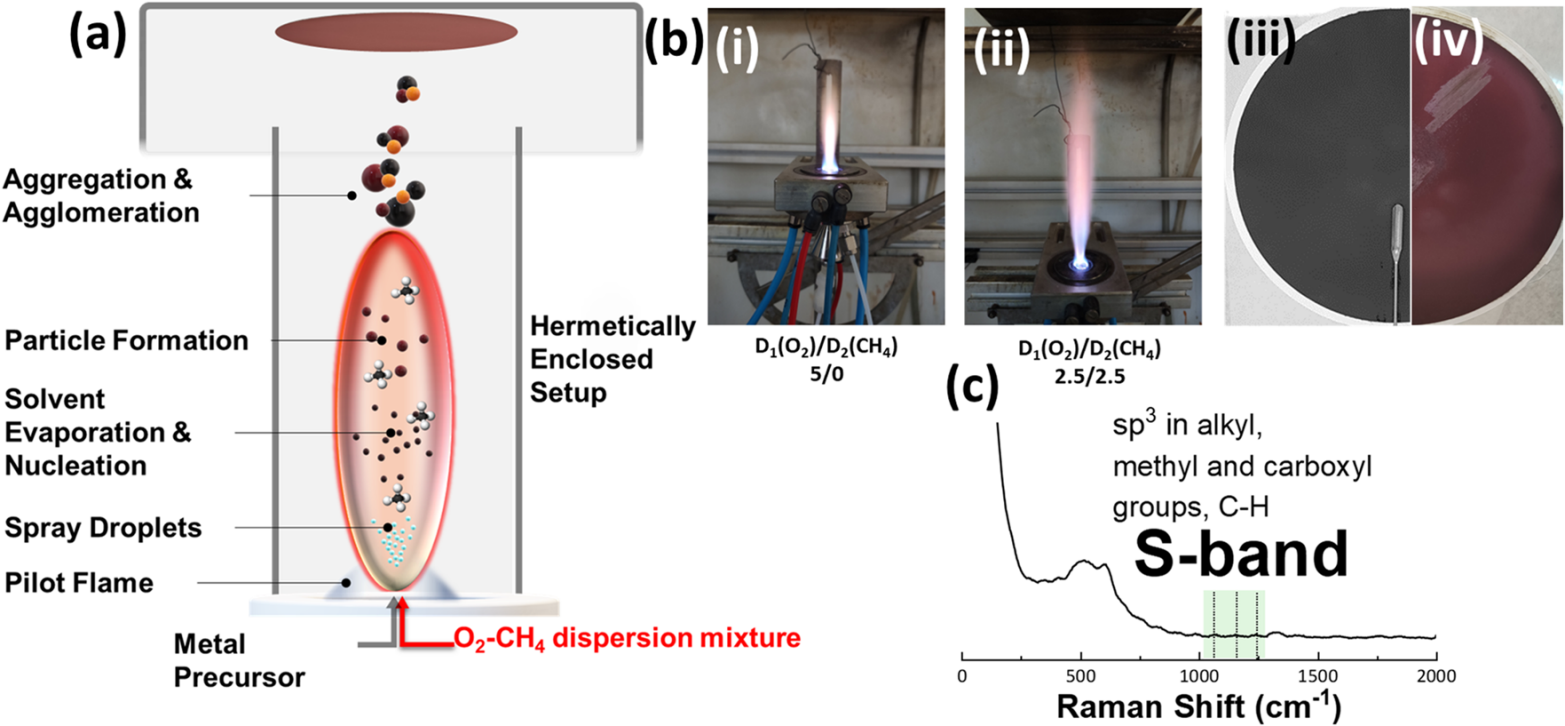
(**a**) Schematic representation of our Anoxic-FSP reactor used for the synthesis of Cu_2_O/Cu^0^ NPs. (**b**) (i)–(ii) Photographs of our nozzle/flame setup in the case of D_1_/D_2_: 5/0 and D_1_/D_2_: 2.5/2.5. (iii)–(iv) Photographs of the particle collection filter. The characteristic black color indicates that the deposited particles are CuO (left) whilst the burgundy-color is characteristic of mixed phase Cu_2_O/Cu^0^ NPs (right). (**c**) Raman spectra of the nanopowder produced with D_1_/D_2_: 1.75/3.25. With green color we highlight the S-band with is attributed to nGC which originate from alkyl, methyl and carboxyl groups.

The anoxic-FSP synthesis of Cu NPs was carried out within an enclosed-flame FSP reactor, with a dispersion feed consisting of {oxygen (O_2_)–methane (CH_4_)} mixture that allows creation of a strongly reducing atmosphere in the flame (Fig. 1b i–ii)^39^. N_2_ sheath gas was used to further maintain the anoxic atmosphere over the flow-field of the particles. A 35 cm metal tube, with no-gap, was used to enclose the flame and to shield the whole flow-field, up to the particle collector system, from ambient air (Fig. 1a). As we have already shown in a previous work^39^ this configuration allows controlled anoxicity, which in the preset cases served a double purpose, see also scheme in Fig. 1a: [i] to promote formation suboxic Cu-phases i.e., Cu_2_O or Cu^0^, [ii] to promote carbon deposition on the Cu_2_O/Cu^0^ nanoparticles, in tandem with their formation. According to the FSP process principles of operation^ 40^ the formation of Cu NPs involves a sequence of key-steps: first the formation of Cu-precursor droplets which contain the fuel (solvent) that evaporates and is combusted, leading to the formation of the primary Cu-particles^40^. The size of primary particles is usually < 1 nm and is controlled by the flow rate of precursor, P, the P/D ratio, and precursor solution composition. Then, these primary particles evolve in the high temperature region of the flame (900–2800 K) and form stable Cu-nanoparticles via sintering of the primary particles^40^, see Fig. 1a. In our A-FSP process, the CH_4_ combustion from the O_2_–CH_4_ dispersion mixture, allows refined control of the synthesis parameters and the composition of the final material. For convenience we codename the O_2_-dispersion feed (lt·min^−1^) as D_1_ and the CH_4_-dispersion feed D_2_ (lt·min^−1^). The controlled transition of phase- composition from CuO to Cu_2_O and finally to Cu^0^ is promoted via decrease of D_1_/D_2_ ratio (Fig. 1b iii–iv). At the same time, the high combustion enthalpy of CH_4_ in D_2_, i.e., 50–55 MJ/kg, increases the combustion temperature, which in turn minimizes the deposition of graphitized carbon. As we verify by Raman (Fig. 1c), under ratios D_1_/D_2_ in the range 5/0 to 1.75/3.25, fully listed in Supplementary Table S1, no graphitized carbon was deposited on the obtained Cu-particles (Fig. 1c). In contrary, the Raman, TGA and FTIR data reveal the deposition of traces of carbonaceous sp^3^ structures marked as S-band (Fig. 1c) which will be discussed later. Moreover, TEM data, confirm the formation of spherical Cu-particles, of controlled phase composition CuO/Cu_2_O or Cu_2_O/Cu^0^ that form heterojunctions.

### Structural characterization

**Figure 2:**
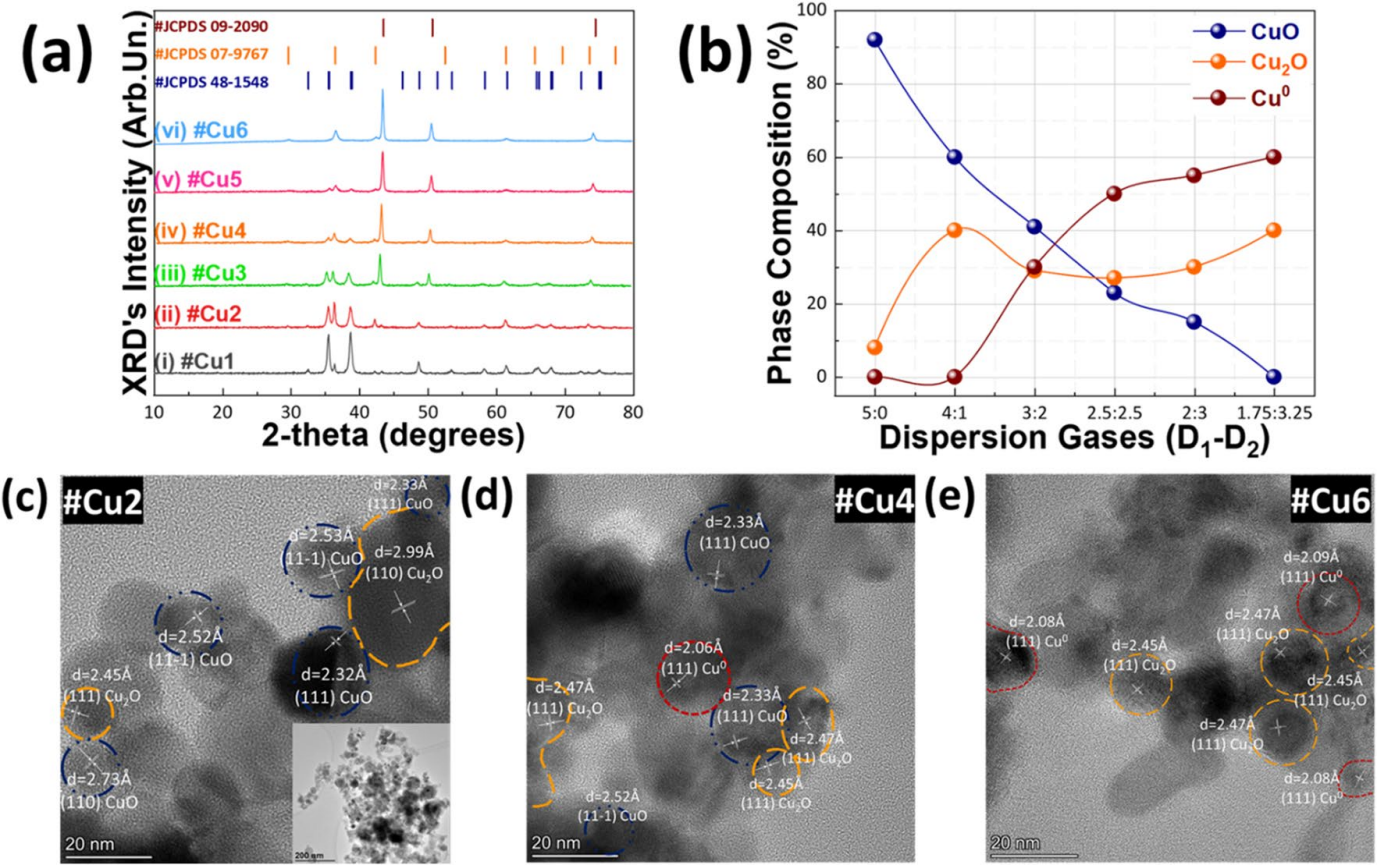
(**a**) XRD patterns of the FSP-made Cu NPs. (**b**) Phase composition (%) diagram versus the different dispersion gases we used for their synthesis. (**c–e**) TEM images of #Cu2, #Cu4 and #Cu6 materials where D_1_/D_2_: 4/1, D_1_/D_2_: 2.5/2.5 and D_1_/D_2_: 1.75:3.25 respectively.

Figure 2a (i–vi) displays XRD data for Cu-particles prepared under different D_1_(O_2_)/D_2_(CH_4_) ratios, see full list of A-FSP parameters in Supplementary Table S1 in S.I. Starting from material #Cu1, i.e., D_1_/D_2_(O_2_/CH_4_):5/0, we have the formation of 92% CuO (JCPDS 48-1548) and 8% Cu_2_O (JCPDS 07-9767). The Cu_2_O-fraction formation is attributed to the slightly anoxic conditions created by the N_2_ sheath^16,39,41^ i.e., since D_2_(CH_4_) = 0 in this case. By changing D_1_/D_2_ to a more reducing profile 4/1, see material #Cu2, we have a decrease of CuO phase percentage, favoring the formation of 40% Cu_2_O. Further increase of D_2_(CH_4_) promotes preferably the Cu^0^ (JCPDS 09-2090) phase percentage, while Cu_2_O phase percentage remains rather constant, and decrease of CuO percentage respectively (Fig. 2b).

TEM images, show that our nanoparticles have well-formed crystal Miller planes, with quasi-spherical shape (Fig. 2c inset) and further verify the phase compositions evidenced by XRD (Fig. 2c–e).

**Figure 3:**
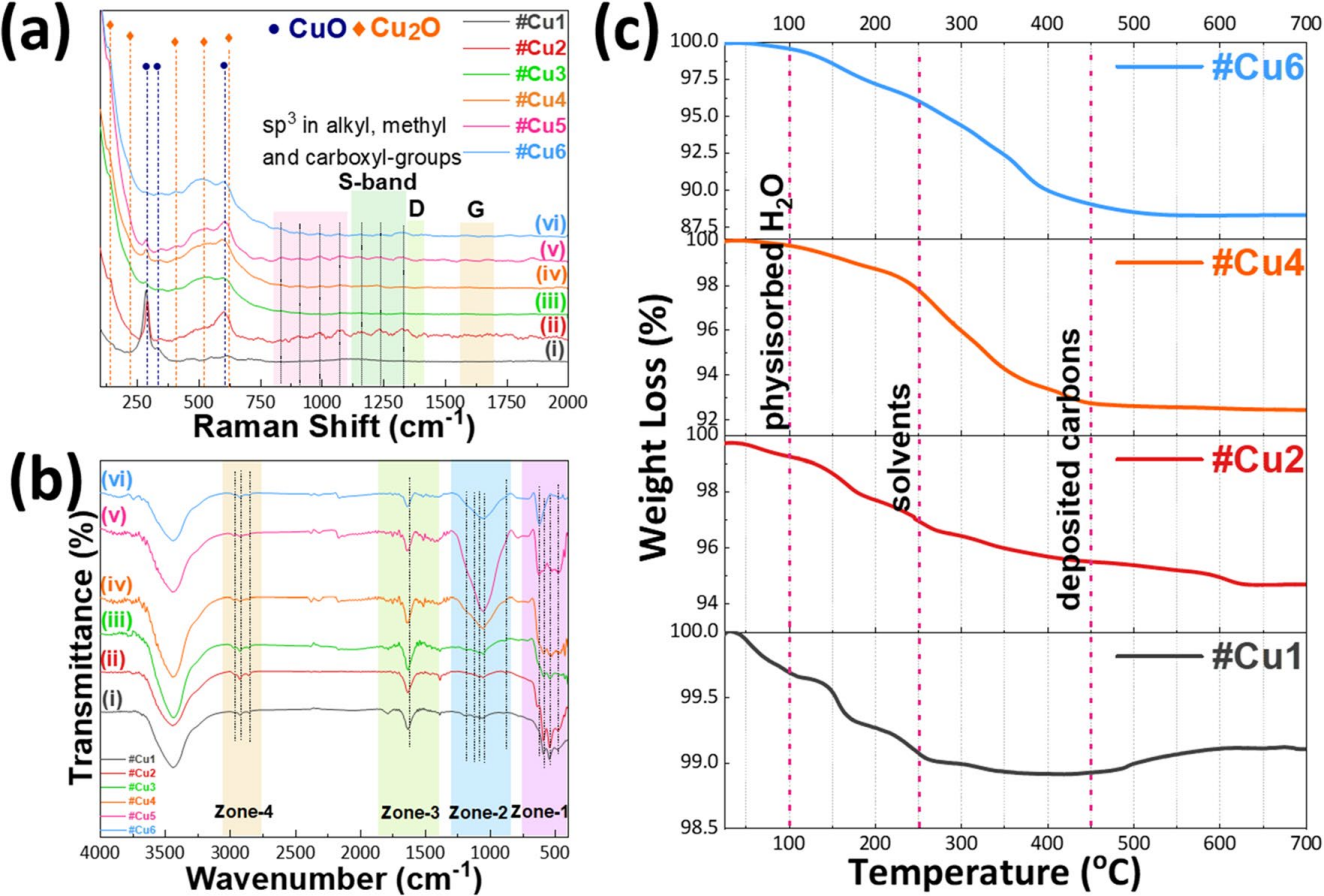
(**a**) Raman and (**b**) FT-IR spectra of the as prepared A-FSP materials. With the different colors we mark the different regions of interest. (**c**) TGA of #Cu1, #Cu2, #Cu4 and #Cu6.

Raman spectra (Fig. 3a) provide detailed information regarding the vibrational modes from both CuO and Cu_2_O nanophases. By definition, metallic phases e.g., Cu^0^, are not detectable by Raman. Thus, the as-prepared Cu NPs exhibited the characteristic Raman bands of CuO or Cu_2_O at 290, 337, 608 cm^−1^ and 132, 220, 410, 523, 620 cm^−1^ respectively (Fig. 3a), summarized in Supplementary Table S2 in S.I. along with reference literature values. In most cases, we observe the characteristic Raman peak of CuO at 290 cm^−1^ which decreases as we increase the D_2_(CH_4_) dispersion i.e., decreasing D_1_(O_2_)/D_2_(CH_4_) ratio (see Fig. 3a (i–v)). Interestingly, the increase of D_2_(CH_4_) content, promotes the formation of Raman bands, marked as S-band in Fig. 3a, while the well-known D–G bands (1350 and 1590 cm^−1^) of graphitized carbon^42^ are absent from the Raman spectra. The S-band Raman peaks, correspond to non-graphitized carbon sp^2^–sp^3^ structures^43,44^ originate from alkyl, methyl, and carboxyl groups^43,44^. In our case, the formation of these non-graphitized carbons is attributed to incomplete combustion of the solvent i.e., acetonitrile-ethylene glycol, due to the oxygen-lean conditions created by the increase of D_2_(CH_4_). Methane itself is unlike to contribute to this non-graphitized carbon i.e., as we verified by control experiment where CH_4_ gas was supplied independently to the flame (data not shown).

These results are further supported by FT-IR spectra (Fig. 3b). We can identify the functional groups present in our materials by classifying the observed peaks to 4 different zones (Fig. 3b). The peaks observed in the range of 400–750 cm^−1^ (Zone-1) correspond to the vibrational modes of Cu–O in the lattices of CuO and Cu_2_O. In the range between 840 and 1300 cm^−1^ (Zone-2) we observe several peaks where the peak centered at 880 cm^−1^ is attributed to the bending vibration of C–H group. The peaks at 1045, 1084, 1123 and 1184 cm^−1^ are assigned to C–O stretching. In zone-3, the peak around 1634 cm^−1^ can be assigned to C=C, and in zone-4 the peaks centered at 2850, 2923 and 2960 cm^−1^ belong to aliphatic CH groups. The strong broad absorption band from 3200 to 3750 cm^−1^ corresponds to hydroxyl (OH) functional groups. For convenience the main FT-IR spectral features are summarized in Supplementary Table S3.

The chemical states of the Cu and C elements were further evaluated by XPS, as shown in Supplementary Fig. S1. XPS of Cu_2p_ (930–970 eV)^45^ can be deconvoluted to six main peaks, by curve fitting. The peaks at 934.3 and 954.8 eV can be assigned to Cu^2+^ (CuO)^45^, while the peaks at 932.6 and 952.6 eV correspond to Cu^1+^ or Cu^0^ (Cu_2_O or Cu^0^)^45,46^ (Supplementary Fig. S1a–c). Carbon peaks are deconvoluted into four peaks where the main peak shown at 284.6 eV corresponds to C–C bond generated by sp^2^ orbital hybridization^47^. The peaks at 286.5, 288.3 and 290.3 eV correspond to C–O, C=O and O=C–O groups respectively^47^ (Supplementary Fig. S1d–f).

From the TG–DTA data, Fig. 3c, we have three regions of interest. The first region exhibits a weight loss at T ≤ 110 °C which corresponds to the desorption of physiosorbed water. In the range of 110–240 °C we observe a second weight-loss attributed to traces of uncombusted solvents which is typical for FSP-made particles^48^. In the third region for temperatures between 240 and 450 °C we have the combustion of deposited non-graphitized carbons (nGC). Typically, in TGA, graphitized carbons deposited on nanoparticles are combusted at T > 450^0^C^49^, thus the present TGA data, corroborate our Raman and FTIR, confirming that the A-FSP process used herein produces exclusively nGC deposited in the Cu-nanophases. For increasing D_2_(CH_4_) in the dispersion feed, we observe an increase in nGC deposition^50^, that accounts for less than 1% in #Cu1 and increased to 7.8% for #Cu6. For comparison, using a more reducing FSP protocol i.e., radial incorporation of acetylene, we have prepared a material, codenamed #Cu-G, with graphitized-carbon characteristics and Cu-phase composition similar to #Cu6, see Supplementary Table S1 and XRD in Supplementary Fig. S2a. The Raman data for #Cu-G, Supplementary Fig. S2b, verify the presence of graphitized carbon i.e., via the typical D and G-bands^42^. Thus, material #Cu-G has enhanced graphited carbon content, in contrast to #Cu2–#Cu6 where graphitized carbon is absent. TG–DTA data (Supplementary Fig. S2c) for #Cu-G show the expected weight-loss for graphitized carbon, extending up to 250–700 °C^49^. This accounts for 13.8% of carbon-content, vs. 7.8% #Cu6. This clearly exemplifies the versatily of A-FSP as a technology can selectively deposit low amounts of nGC or higher amounts of more-graphitized carbon on the Cu-nanophases.

### Photocatalytic performance

**Figure 4:**
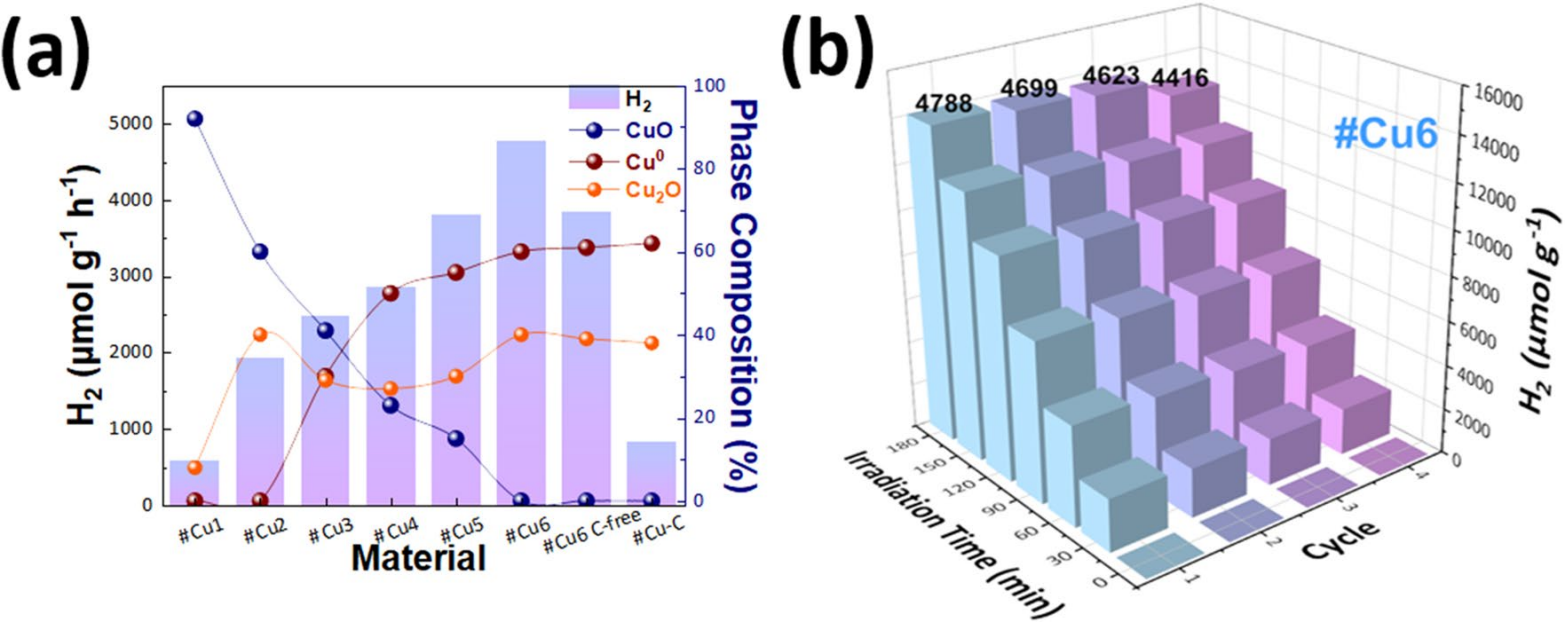
(**a**) Photocatalytic H_2_ production from H_2_O of the Cu NPs under, xenon-light irradiation. (**b**) Consecutive reuse of #Cu6 material for H_2_ production.

Figure 4a presents rates of photocatalytic H_2_ production from a H_2_O/CH_3_OH = 80/20 mixture, under simulated solar-light irradiation, Xenon, for the #Cu-catalysts. The reference material #Cu1 achieved the lowest H_2_ production yield 580 μmol g^−1^ h^−1^, which is consistent with the fact that it consists of mainly CuO phase. Increase of D_2_(CH_4_) i.e., increase of Cu_2_O and Cu^0^ phase-percentages at the expense of CuO, boosts the H_2_ production with the material #Cu6 having the highest H_2_ production of 4788 μmol g^−1^ h^−1^, with an Apparent Quantum Yield (AQY) 0.55% at λ = 440 nm. Interestingly, upon removal of the nGC (see Supplementary Fig. S3 in S.I.) via consecutive washings with DMF, while maintaining the same phase composition, the so-obtained {#Cu6 C-free} material showed a noticeable decrease in H_2_ production to 3850 μmol g^−1^ h^−1^. Moreover, we have verified, by FTI-IR, that the #Cu6 C-free contained no traces of DMF (data not shown). Therefore, we can conclude that the presence of the nGC has a beneficial effect on the overall H_2_ production. Finally, the graphitized-C material #Cu-G, achieved inferior photocatalytic H_2_ production of ~ 800 μmol g^−1^ h^−1^. This, negative effect of GC can be attributed to two main effects (i) the high C-loading in #Cu-G, may block the Cu-catalyst surface-sites, in contrast the nGC—by its nature/lower content/lower coating-capacity does not suffer from this drawback. (ii) Graphited Carbon in #Cu-G may act as an electron-sink, competing with the H_2_-forming process. A key-observation is that our catalyst with the highest H_2_ yield, #Cu6, was highly recyclable retaining > 95% of its activity after 4-uses (Fig. 4b). Moreover, post catalytic characterizations indicate that this material exhibits largely unaltered phase-composition even after 4 catalytic cycles (see XRD, Raman, FTIR data in Supplementary Fig. S4a–c). More specifically, the XRD data verify the Cu_2_O/Cu^0^ stability, while Raman and FT-IR data show that nGC moieties remain unaltered. This stability stems inherently from the FSP process i.e., the concomitant formation of the Cu-particles and the nGC in the high-temperature regime of the flame, promotes tight the {nGC-Cu-particle} association which in turn ensures stability under the liquid-phase photocatalytic process.

Thus, the present data demonstrate that (i) optimization of the phase composition of Cu_2_O/Cu^0^ nanohybrids is key-condition for enhanced H_2_ photoproduction, (ii) the presence of nGC has a beneficial effect on the H_2_ photoproduction by Cu_2_O/Cu^0^ nanohybrids, and nGC is superior vs. more-graphitized carbon, (iii) the beneficial role of nGC correlates to reusability of the catalysts, indicating that is linked to minimization of its photocorrosion, as we verify in the following by photoelectrochemical analysis.

### Photoelectrochemical (PEC) study

**Figure 5:**
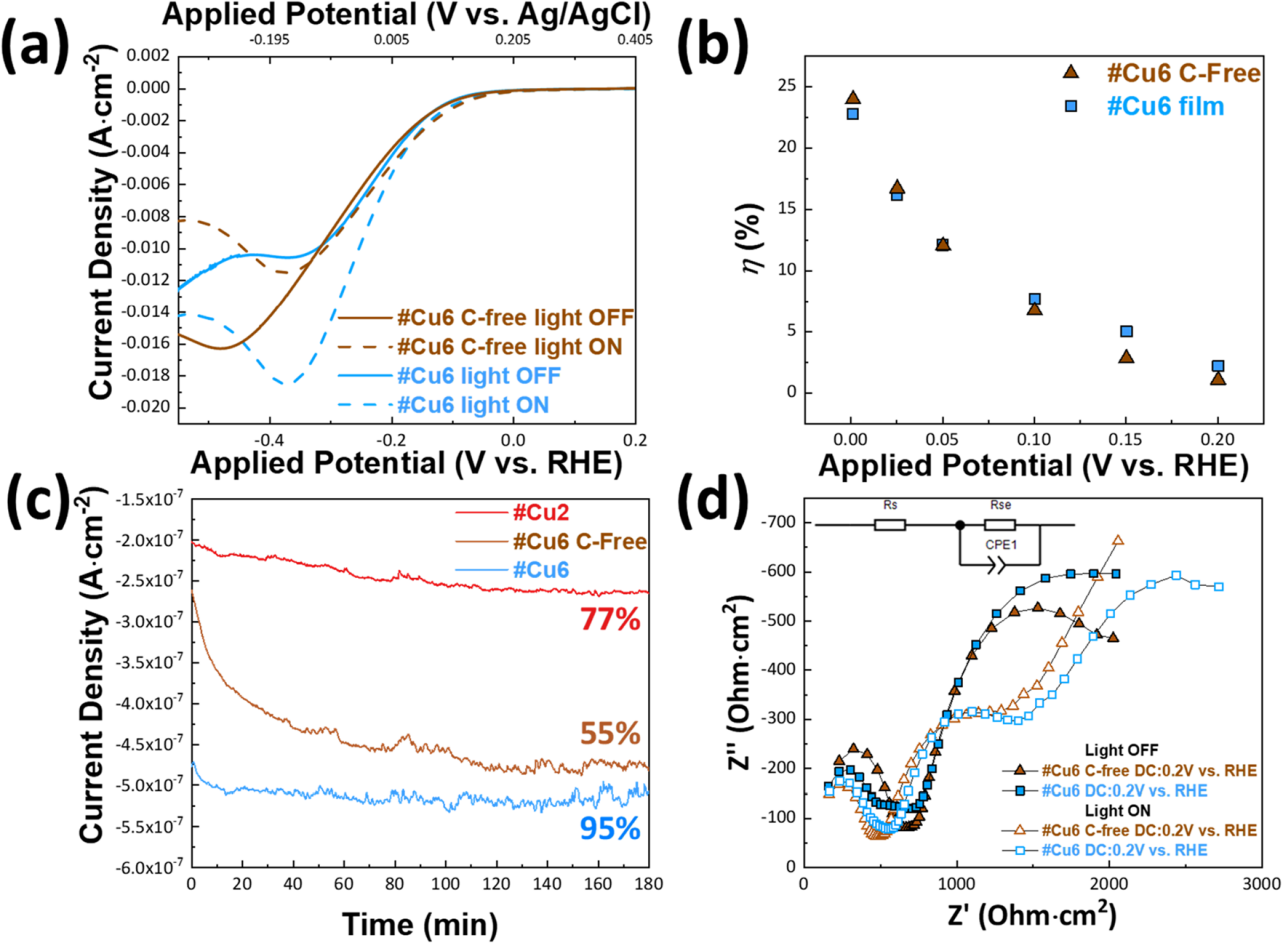
(**a**) [Current Density] vs. [cathodic potential] data for #Cu6 C-free film (brown line) and #Cu6 film (blue line), under dark (solid lines) and under continuous irradiation (dashed lines). (**b**) [Applied potential] vs. [photo-to-current conversion efficiency (*η*) (%)] for #Cu6 C-free (brown line) or #Cu6 (blue line) (**c**) Stability of #Cu6 C-free film (brown line) and #Cu6 film (blue line) in 0.1 M NaOH (**d**) Nyquist plot of the #Cu6 C-free film (brown line) and #Cu6 film (blue line), photocathodes under dark condition (solid symbol) and under continuous irradiation (open symbols).

In Fig. 5a, PEC current versus voltage data (i.e., LSV—Linear Sweep Voltammetry) are presented for the catalysts, either in dark condition (“light OFF”) or under illumination at a selected wavelength of 455 nm and light intensity of 133 mW cm^−2^ (“light ON”). For comparison in Fig. 5a, we show LSVs for the #Cu6 and “#Cu6-C free” photocathode-films, in 0.1 M NaOH at pH = 13.4. For completeness, LSV data for materials #Cu3–5 which also show increased H_2_ production, are presented in Supplementary Fig. S5a of the Supporting Information.

At 0 V vs. RHE, under dark (light-Off), both films exhibit similar current density, i.e., J = − 0.12 mA cm^−2^ and J = − 0.11 mA cm^−2^ the for {#Cu6 C-free} and #Cu6-film respectively, see Fig. 5a. Under light-On, #Cu6 C-free and #Cu6 films achieved comparable photocurrent densities of J = − 0.20 mA cm^−2^ and J = − 0.19 mA cm^−2^ respectively. All these data are summarized in Supplementary Table S4. The photocurrent onset-potential of #Cu6 C-free film was 0.188 V vs. RHE under light-On, and 0.179 V vs. RHE in dark. Importantly, the photocurrent onset-potential of #Cu6 film was 0.212 V and 0.234 V vs. RHE, in dark and light conditions respectively. Thus, this comparison shows that the nGC on #Cu6 film, is beneficial to achieve a better onset potential, in both dark and light. Under dark, the overpotential of #Cu6 C-free and #Cu6 films, at J = 10 mA cm^−2^^51^, were comparable i.e., − 0.320 V for #Cu6 and − 0.319 V for #Cu6 C-free. However, under continuous irradiation, i.e., #Cu6 film showed an exceptional overpotential of − 0.252 V vs. RHE while #Cu6 C-free film had an overpotential at − 0.307 V vs. RHE, see Fig. 5a. The positive difference of 55 mV ensures a much-lower reductive decomposition rate of copper oxides during PEC HER, which benefits the stability of photocathode and might lower the photocorrosion^52,53^.

To estimate the performance of the photoelectrodes under-bias^54–56^, the {applied potential photon to current density} efficiency (*η*) was calculated via Eq. (1).

1 $$\eta \left(\%\right)={J}_{p}\frac{\left(1.23-\left|{E}_{ocp}-{E}_{app}\right|\right)}{P}\times 100\%,$$

where *J*_*p*_ is the photocurrent density (mA cm^−2^), *E*_*ocp*_ is the open circuit potential^54–56^ in the same solution and under the same radiation of light, *E*_*app*_ is the applied potential at which *J*_*p*_ was measured^54–56^ and *P* is the incident light power density (mW cm^−2^), as we measured it in-situ by the power meter. In Fig. 5b**,** *η* was estimated in 0.1 M NaOH, under a light-power density of 133 mW mW cm^−2^. Under these conditions, *E*_*ocp*_ values for #Cu6 C-free and #Cu6 films were 0.041 V and 0.080 V vs. RHE, respectively. As reference, at 0 V vs. RHE, both films achieved comparable *η* efficiencies near 17.5%. Under positive-bias, +0.05 V vs. RHE, both films achieved a conversion efficiency of *η* = 0.08%. Under a bias + 0.15 V vs. RHE, #Cu6 achieved *η* = 0.04% vs. *η* = 0.02% for #Cu6 C-free film.

An important factor is the photostability of these photocathodes, especially for long-term PEC HER. As shown in Fig. 5c, the #Cu6 C-free film shows a low photostability of 55% in the first 120 min, indicating a high-decay of 45%. In contrast, #Cu6 showed a considerable stability of 95%, with fast stabilization response of only 16 min see Fig. 5c. Moreover #Cu6 was stable after 3 h of illumination and applied potential of 0V *vs* RHE, with a limited decay of 5%. Thus, it can be concluded that the #Cu6 film which consists of (Cu_2_O and Cu^0^) phases, thanks to the presence of non-graphitized carbon, gained high photostability against photocorrosion, as evidenced by direct comparison with the very unstable #Cu6 C-free film with the same phase composition.

Additionally, to clarify the origin of high photocurrent density, Electrochemical Impedance Spectroscopy (EIS) was employed to investigate the charge transfer rate between the interface of semiconductor and solution. In Fig. 5d we present the Nyquist plots of the two photocathodes (#Cu6 C-free and #Cu6 films) at 0 V vs. RHE in 0.5M Na_2_SO_4_ (pH = 6.0) in dark and under continuous irradiation conditions. Nyquist plots for #Cu3-5 are also presented in Supplementary Fig. S5b. Moreover, for #Cu6 C-free and #Cu6 films, the corresponding plots at 0V vs. RHE in 0.1 M NaOH (pH = 13.4) under light-On conditions, are presented in Supplementary Fig. S6. In brief, in Nyquist plots, the semicircle at low-frequencies, is determined by the charge transfer phenomena across the semiconductor/electrolyte interface and the diameter of the semicircle represents the charge transfer resistance (R_se_)^57^. The calculation of R_se_ value is achieved with the use of an equivalent circuit, see inset in Fig. 5d. In Supplementary Table S5 we present the component values derived by the fit of equivalent circuit. The R_se_ values for different applied DC potential values presented in Supplementary Fig. S7 in Supplementary information. According to Fig. 5d**,** and Supplementary Fig. S7, under continuous irradiation, R_se_ is decreasing for both photoelectrodes, which indicates that illumination accelerates the charge transfer phenomena at the semiconductor/solution interface, due to the photoinduced increase of carrier density^57^. More specifically, #Cu6 C-free film, under dark conditions and a DC potential of 0.2 V vs. RHE, had R_se_ of 5.19 kΩ cm^−2^ while under illumination R_se_ was lower 4.6 kΩ cm^−2^. Under the same conditions, the #Cu6 film, achieved lower resistance R_se_ of 4.92 kΩ cm^−2^ and R_se_ of 3.9 kΩ cm^−2^ under dark and continuous irradiation conditions, respectively. As presented in Fig. 5b both photoelectrodes present an optimal photon to current density at 0 V vs. RHE, therefore EIS was deployed also to that potential (DC 0 V vs. RHE), Fig. 5d. Accordingly R_se_ (at 0 V) under dark was 38.4 KΩ cm^−2^ for #Cu6 C-free, vs. R_se_ 38.6 KΩ cm^−2^ for #Cu6. Under illumination, R_se_ of the #Cu6 film was 19.8 KΩ cm^−2^ that is significantly lower than R_se_ = 28.2 KΩ cm^−2^ for #Cu6 C-free. Overall, these data demonstrate that, while under dark conditions the presence of nGC makes no difference in the R_se_ value, under continuous light irradiation the presence of nGC significantly decreases the resistance R_se_ in #Cu6.

## Discussion

In our recent work^39^, we have exemplified the Anoxic-FSP process as a tool to engineer ZrO_2−x_ can with high-photocatalytic performance by controlling the population of cluster-Vo’s versus monomer-Vo’s^39^. In the case of ZrO_2_, zero-carbon, neither graphitized or non-graphitized, was deposited on the final material. In the present work, use of A-FSP allows controllable deposition of non-graphitized carbon on {nGC/Cu_2_O/Cu^0^} phases. The present data provide clear evidence that presence of nGC in the Cu_2_O/Cu^0^ nanohybrids: (i) acts beneficially to the photocatalytic H_2_ via improving the mobility of photoinduced electrons-holes, that is the key-beneficial factor for the observed high H_2_-photogeneration performance of this {nGC/Cu_2_O/Cu^0^} catalyst. (ii) improves stability against photocorrosion, that is the key-beneficial factor for the observed reusability for this {nGC/Cu_2_O/Cu^0^} catalyst.

To further understand the dynamics of the photoinduced carriers, were further investigated the majority carrier density of the copper photoelectrodes {nGC/Cu_2_O/Cu^0^} photoelectrodes by Mott–Schottky analysis^58–60^. In brief, according to Mott–Schottky theory^58^, Eq. (2), the space-charge capacitance (C_sc_) of semiconductors varies as a function of applied potential.

2 $$\frac{1}{{C}_{sc}^{2}}=\frac{2}{e\varepsilon {\varepsilon }_{0}{\rm N}_{\rm A}}\left({E}_{app}-{E}_{fb}-\frac{{k}_{B}T}{e}\right),$$

where *e* is the electron charge, *ε*_*0*_ is the permittivity of the vacuum, *ε* is the dielectric constant of the semiconductor (for Cu_2_O, *ε* is 7.6)^61–63^, *N*_*A*_ is the acceptor density (majority carrier density, i.e. hole density in *p*-type semiconductors such as Cu_2_O), *E*_*app*_ is the applied potential, *E*_*fb*_ is the flat band potential, *k*_*B*_ is the Boltzmann’s constant, and *T* is the absolute temperature. Thus, by using Eq. (2), the flat-band potential, E_fb_, can be estimated, as well as the majority carrier density *N*_*A*_ of the semiconductor.

**Figure 6:**
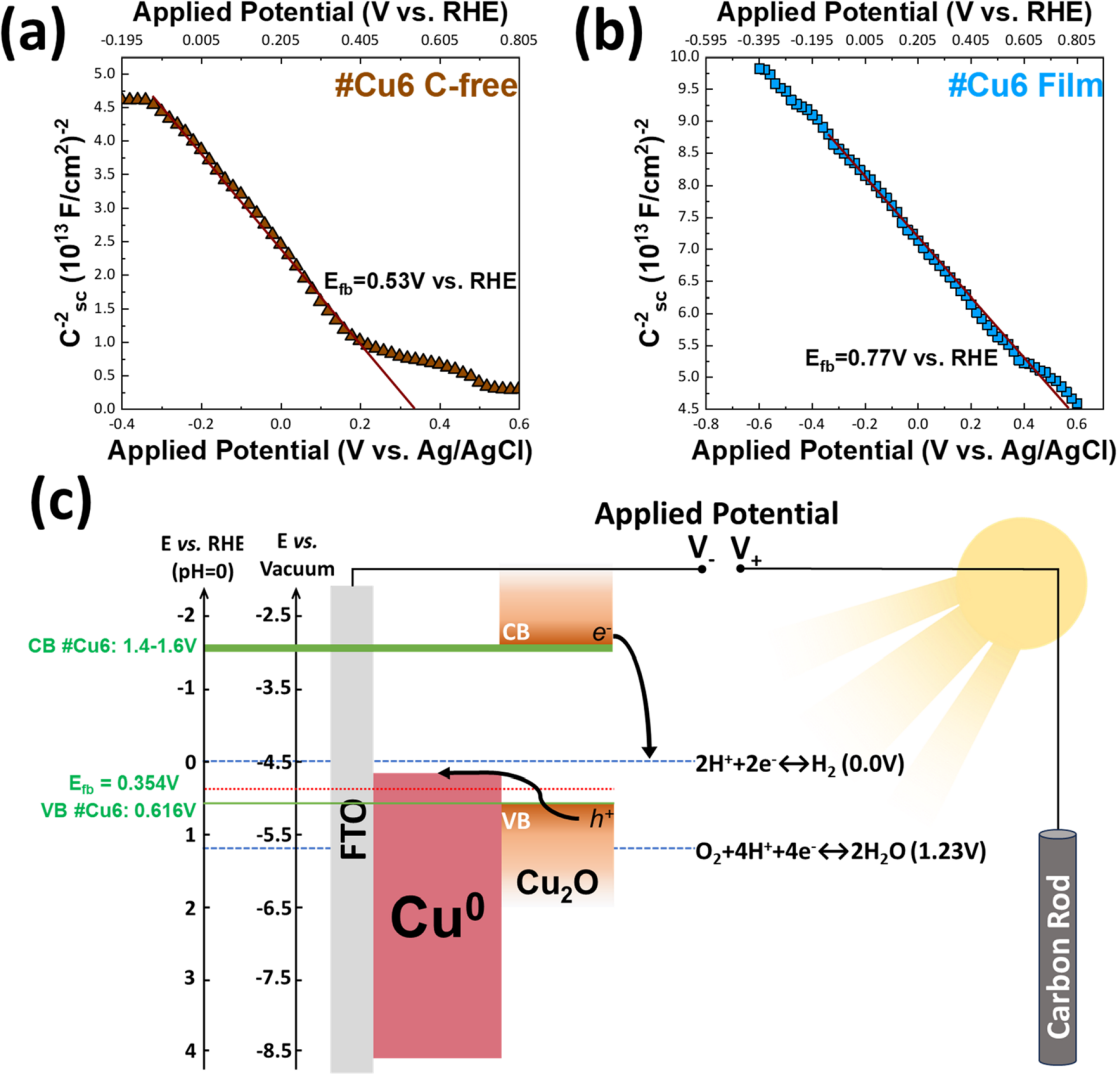
Mott-Schottky plots, and flat band potential estimation under continuous illumination (visible light 455 nm, 133 mW cm^−2^). (**a**) #Cu6 C-free film (brown symbols) (**b**) #Cu6 film (blue symbols). The solid lines in (**a**,**b**) are fits of the Mott–Schottky Eq. (2) (**c**) Schematic representation of the energy band diagram of #Cu6 in contact with solution during PEC HER.

Figure 6a,b show the Mott–Schottky plots of #Cu6-C free and #Cu6 photoelectrodes, under visible light 455 nm, 133 mW cm^−2^. The negative slopes indicate that they both are *p*-type semiconductors. Fitting Eq. (2), to the data in Fig. 6a,b, see solid-lines, the majority carrier density (N_A_), calculated for Cu6-C free N_A_ was 4.58 × 10^15^ a cm^−3^, vs. N_A_ 5.4 × 10^15^ a cm^−3^ for #Cu6. Higher N_A_ signifies fast charge-transfer, thus enhanced PEC performance. From the X-intercept in Fig. 6a,b, the flat-band potential is estimated E_fb_ = 0.53 V for #Cu6-C free, and E_fb_ = 0.77 V for #Cu6. For *p*-type semiconductors, higher E_fb_ value indicates a higher degree of band-bending, and a larger space-charge-region potential^60,61^. Thus, the higher E_fb_ value of #Cu6 provides more efficient photo-induced electron–hole separation in the space charge region, i.e., a higher photoactivity for Hydrogen Evolution Reaction (HER).

It is well-known that when a semiconductor/solution interface is under intense illumination, the band bending energy is minimized^64^, thus both the conduction and valence bands are flat, and the photocurrent onset potential approaches E_fb_^64^. Under this condition, the E_fb_ is equal to Fermi energy E_F_. Using this method, the valence band-edge E_V_ can be obtained by Eq. (3) ^58^.

3 $${E}_{V}={E}_{F}+\frac{{k}_{B}T}{e}ln\frac{{N}_{V}}{{N}_{A}},$$

where E_F_ is the Fermi level in Volts. N_V_ is the effective density of states in the valence band^58,65^ that can be estimated by Eq. (4).

4 $${N}_{V}=2\frac{{(2\pi {m}^{*}{k}_{B}T)}^{3/2}}{{h}^{3}},$$

where the effective mass m* of the holes was set to 0.58m_0_ for Cu_2_O and m_0_ as the mass of the free electron^64,65^. Thus, using Eqs. (3) and (4), for Cu_2_O the value of N_V_ was found to be 1.11 × 10^19^ cm^−3^. Accordingly, the valence band edge (at pH = 6.0) is estimated to be 0.97V for #Cu6 *vs.* 0.73 V for #Cu6-C free, photoelectrode. By extrapolation at pH = 0, the valence band edge is estimated E_VB_ = − 5.1 eV (#Cu6) and E_VB_ = − 4.88 eV (#Cu6-C free). For a band gap of Cu_2_O 2.0–2.2 eV, the conduction band edges for the two photoelectrodes can be calculated to E_CB_ = − 3.1–2.9 eV for #Cu6, and E_CB_ = − 2.87–2.67 eV for #Cu6-C free. All these data can be visualized in Fig. 6c. This indicates #Cu6 has a more negative E_CB_, that is beneficial for the photoreduction mechanism of H_2_ production.

Overall, herein we have developed an FSP method for controlled synthesis of Cu-nanophases, interfaced with carbons. We found that, nGC/Cu^2^O/Cu^0^ nanohybrids containing non-graphited carbon at low quantities, consist of a novel-type of Cu-photocatalysts with enhanced H_2_-photocatlytic production, intimately linked with improved photo stability.

## Methods

### Synthesis of nanomaterials by FSP

The CuO/Cu_2_O/Cu^0^ nanomaterials were produced in enclosed single nozzle FSP reactor. The spray nozzle was enclosed by a 20 cm cylindrical metal chamber excluding the environmental oxygen from the flame. The O_2_-controlling system consists of two concentric tubes. The outer tube is a sinter metal tube whereas the inner tube is a perforated metal tube. A precursor solution of 0.25 M was prepared by dissolving Copper (II) Nitrate trihydrate (99–104%, Aldrich) in a 1:1 (by volume) mixture of acetonitrile [≥ 99.9%, Supelco (Bellefonte, Pennsylvania, USA)] and ethylene glycol [≥ 99%, Supelco (Bellefonte, PA, USA)]. This solution is fed into the FSP burner though a capillary at a feed rate of 3 mL min^−1^ and atomized into fine droplets using a dispersion flow rate of 5 mL min^−1^. The resulting spray was ignited and sustained by an oxygen/methane pilot flame (O_2_ 2 L min^−1^, CH_4_ 1.2 L min^−1^). Another important characteristic of our lab-scale FSP reactor is the modified dispersion feed. Keeping a constant dispersion feed rate at 5 mL min^−1^, CH_4_ was also used as a dispersion gas along with the traditional O_2_. This modification of the dispersion results in higher temperatures and formation of reducing agents which allows us to explore the phase transformation from CuO to Cu_2_O and Cu^0^. Finally, for the particle collection an additional 10 L min^−1^ N_2_ sheath was used. The produced nanoparticles were deposited on a glass microfiber filter (Hahnemühle GF 6 257) with the assistance of a vacuum pump (BUSCH V40).

### Characterization techniques

#### Powder X-ray diffraction (pXRD)

The as prepared CuO/Cu_2_O/Cu^0^ nanomaterials were characterized using a powder X-ray diffractometer (Bruker D8 Advanced using CuK_α_ radiation = 1.5405 Å) with a scanning step of 0.03° at a rate of 2 s per step and 2-theta (*θ*) angle ranging from 10° to 80° at current 40 mA and voltage 40 kV. The average crystal size was calculated by using the Scherrer equation. To determine the percentage of CuO/Cu_2_O/Cu^0^ crystal-phase in each Cu-based nanomaterial we used Profex which is a graphical user interface for Rietveld refinement.

#### Fourier transformed infrared (FT-IR)

FT-IR spectra were collected using an IR Nicolet IS5 system equipped with the OMNIC software package in the wavenumber range of 400–4000 cm^−1^ for materials dispersed in KBr pellets.

#### Raman spectroscopy

Raman spectra were obtained with a HORIBA-Xplora Plus spectrometer, equipped with an Olympus BX41 microscope. A 785 nm diode laser was used as an excitation source, and the laser beam was focused on the sample with the aid of the microscope. Before measurement, each powder material dispersed in KBr which serves as an excellent heat-dissipating medium to prevent heating of the sample and potential phase alteration. Raman spectra were recorded performing 50 accumulations in 5 s at fixed low intensity i.e., 1% of the total intensity of the laser, in which the crystal phase of our material remained unchanged, and the signal-to-noise ratio was satisfactory.

#### Thermogravimetric analysis (TGA)

Thermogravimetric analysis was carried out by a Setaram Labsys™ Evo instrument using a heat rate of 2 °C min^-1^ from 25 to 700 °C and a flow rate of nitrogen carrier gas of 20 mL min^−1^.

#### X-ray photoelectron spectroscopy (XPS)

XPS data were collected by a surface analysis ultrahigh vacuum system (SPECS GmbH) equipped with a twin Al–Mg anode X-ray source and a multichannel hemispherical sector electron analyzer (HSA Phoibos 100). The base pressure was 2 − 5 × 10^−9^ mbar. A monochromatized Mg Kα line at 1253.6 eV and analyzer pass energy of 20 eV were used in all XPS measurements. The binding energies were calculated with reference to the energy of C_1s_ peak of contaminant carbon at 284.5 eV. The peak deconvolution was calculated using a Shirley background.

#### Transmission electron microscopy (TEM)

TEM: The morphology of the materials was analyzed by transmission electron microscopy using a FEI Titan 80–300 S/TEM microscope at 300 kV accelerating voltage and a 21.5 mrad beam convergence angle. Before the measurements, the nanopowders were dispersed in ethanol, sonicated at a bath sonicator and then deposited as single droplet of suspension on a copper TEM grid covered with a thin carbon layer. Selected area electron diffraction patterns were acquired with the same instrument in TEM mode, under parallel electron beam illumination.

#### Photocatalytic H_2_ production procedure

Photocatalytic hydrogen reactions were performed into an immersion-well quartz reactor cooled with tap circulation (T = 25 °C). As light source a solar simulator (Sciencetech, Class AAA, model SciSun-150) with average irradiation intensity of 180 W m^−2^ equipped with a xenon lamp of 150 W and Air Mass filter (1 sun, AM1.5G). For each experiment 50 mg of catalyst were suspended into 150 mL water/methanol mixture 20% v/v (final concentration 330 mg L^−1^). For the calculation of Apparent Quantum Yield (AQY)%, a Phoseon 6W Led lamp FireEdge™ FE410 (λ = 440 nm) was used, with power intensity set to 53.8 mW cm^−2^, measured in-situ by a digital power meter (Newport, USA). For full detail of AQY, see Eqs. (1), (2) in S.I. Atmospheric O_2_ from the suspension was removed by purging the reactor with Ar gas (99.9997%) for at least 1 h. In-situ photodeposition of Pt-cocatalyst was implemented to increase the photocatalytic production using as precursor dihydrogen hexachloroplatinate (IV) hydrate complex (H_2_Pt_4_Cl_6_·6H_2_O, 99.99%, Αlfa Αesar). Qualitative and quantitative monitoring of produced H_2_ and CO_2_ gases was done via a continuous online GasChromatography System combined with a Thermo-conductive Detector (GC-TCD- Shimadzu GC-2014, carboxen 1000 column, Ar carrier gas).

#### Post-FSP removal of nGC

To remove the nGC from our materials a washing and drying has been followed. Our materials were dispersed in *N*,*N*-dimethylformamide (DMF), sonicated at a bath sonicator for 10 min and a series of washings with ethanol and acetone was followed. This process was repeated multiple time to ensure the removal of the nGC. Finally, we dry our material at 60 °C overnight.

### Film preparation

#### Chemicals and materials

All chemicals were of analytical reagent grade and used without further purification. The fluorine-doped tin oxide (FTO) glass (7 Ω sq^−1^, transparency 80%, Sigma Aldrich, USA) was used as the conductive substrate. All aqueous solutions were prepared with triple distilled water by Millipore.

#### Preparation of #Cu 1–6 photoelectrodes

The #Cu1–6 films were prepared by spin coating method. At first a slurry was prepared by adding 15 mg of #Cu 1–6 catalysts in a mixture of 1.37 mL triple distilled water (Millipore SIMS600 CP Burlington, USA) at room temperature, T = 23 °C, plus 1.63 mL of isopropanol (Merck, ACS Reag, New Jersey, USA). Then The catalyst mixture was ultrasonicated for 20 min, using a 20 W ultrasonication bath (Elmasonic S10 h, Singen, Germany) to achieve a homogeneous slurry. When homogeneity achieved 10 μL of Nafion solution-mixture was added in the mixture and again ultrasonicated for another 20 min to achieve the final catalyst ink. The Nafion-solution consisted of 5 wt% perfluorinated Nafion resin solution (Sigma Aldrich, MO, USA), in triple distilled water/isopropanol (110 μL Nafion solution: 5.5 mL triple distilled water: 6.5 mL isopropanol).

Deposition of each catalyst ink on the Fluorine doped Tin Oxide (FTO) glass which was used as working electrode was performed by spin coating of the prepared homogeneous suspension onto the FTO glass. The film preparation was performed on a SCS 6808P. (Specialty Coating Systems, Indianapolis, USA). The thickness of the #Cu 1–6 films was controlled by determining the spinning speed (rounds per minute). The speed of rotation for reliable and consistent films was selected to be 5000 rpm for 120 s per drop, in total 50 layers were created.

#### Electrochemical and photoelectrochemical measurements

All electrochemical and photoelectrochemical (PEC) measurements were performed on a Cortest CS2350 electrochemical workstation in a three-electrode cell with #Cu1–6 films to act as working electrode, a carbon rod and an Ag/AgCl as counter and reference electrodes, respectively.

The photocurrent was measured under continuous irradiation using a 6W Phoseon FJ100 UV LED source, which emits at 455 nm wavelength, and the intensity of the light source was calibrated with a 1918-C optical power meter (Newport, USA) to simulate AM 1.5 illumination (133 mW cm^−2^).

The area of all photocathodes exposed to light was 15 cm^2^. The liquid solutions used for the PEC activity and stability tests,  were nitrogen-purged, containg either 0.1 M NaOH, pH adjusted to 13.4 or 0.5 M Na_2_SO_4_ at pH v 6.0. Before PEC measurements, the solution was purged with (50 mL/min) N_2_ for 30 min to remove O_2_. Unless indicated, all the potentials were calibrated vs. reversible hydrogen electrode (RHE) according to the following equation:

$${E}_{(RHE)}={E}_{ag/AgCl}+0.059pH+{E}_{Ag/AgCl}^{o},$$

where ${E}_{Ag/AgCl}^{o}=0.1976 V$ at 25 °C and ${E}_{ag/AgCl}$ is the working potential.

Electrochemical impedance spectroscopic measurements were carried out in the dark and under illumination at an AC voltage of 10 mV with a frequency region ranging from 0.01 Hz up to 100 kHz, at three different applied DC potential values. The Mott–Schottky plots were obtained at a frequency of 1 kHz and an amplitude of 10 mV to determine the flat-band potential.

### Supplementary Information

Supplementary Information.

## Data Availability

All data generated or analyzed during this study are included in this published article [and its supplementary information files].
