# Supplementary material for: Non-graphitized carbon/Cu2O/Cu0 nanohybrids with improved stability and enhanced photocatalytic H2 production
Source: Sci Rep. 2023 Aug 26;13:13999. doi: 10.1038/s41598-023-41211-4 (PMC10460407; doi:10.1038/s41598-023-41211-4)
Supplement: Supplementary file 1 — Supplementary Information. [file 41598_2023_41211_MOESM1_ESM.docx]

Supplementary Information for

Non-Graphitized Carbon/Cu_2_O/Cu^0^ Nanohybrids with Improved Stability and Enhanced Photocatalytic H_2_ Production

Areti Zindrou^1^, Loukas Belles^1^, Maria Solakidou^1^ Nikos Boukos^2^ and Yiannis Deligiannakis^1^*

^1^Laboratory of Physical Chemistry of Materials & Environment, Department of Physics, University of Ioannina, Ioannina, Greece

^2^Institute of Nanoscience and Nanotechnology (INN), NCSR Demokritos, 15310 Athens, Greece

* Corresponding author: Yiannis Deligiannakis ([ideligia@uoi.gr](mailto:ideligia@uoi.gr))

**Contents**

[**Supplementary Tables** 2](#_Toc143606527)

[**Table S1: Summarized FSP-process condition for the synthesis of {nGC/Cu_2_O/Cu^0^} nanohybrids** 2](#_Toc143606528)

[**Table S2: Summarized Raman band frequencies of CuO and Cu_2_O** 3](#_Toc143606529)

[**Table S3: Summarized absorption peaks of FT-IR spectra** 4](#_Toc143606530)

[**Table S4: Summarized PEC analysis for #Cu6 C-free and #Cu6 photocathodes films under dark conditions and continuous irradiation (wavelength: 455 nm, 1333 mW**·**cm^-2^) in 0.1M NaOH.** 5](#_Toc143606531)

[**Table S5: Summarized component values for equivalent circuit (simulation) of #Cu6 C-free and #Cu6 in different medium** 6](#_Toc143606532)

[**Supplementary Figures** 7](#_Toc143606533)

[**Supplementary Figure 1** 7](#_Toc143606534)

[**Supplementary Figure 2** 8](#_Toc143606535)

[**Supplementary Figure 3** 9](#_Toc143606536)

[**Supplementary Figure 4** 10](#_Toc143606537)

[**Supplementary Figure 5** 11](#_Toc143606538)

[**Supplementary Figure 6** 12](#_Toc143606539)

[**Supplementary Figure 7** 13](#_Toc143606540)

[**Apparent Quantum Yield (AQY%) Calculation** 14](#_Toc143606541)

[**Supplementary References** 15](#_Toc143606542)

## **Supplementary Tables**

### **Table S1: Summarized FSP-process condition for the synthesis of {nGC/Cu_2_O/Cu^0^} nanohybrids**

| **Material** | **P/D** | **D_1_ (O_2_)** | **D_2_ (CH_4_)** | **Sheath N_2_ (lt min^-1^)** | **Radial Acetylene (lt min^-1^)** |
| --- | --- | --- | --- | --- | --- |
| #Cu1 | 3/5 | 5 | 0 | 10 | - |
| #Cu2 | » | 4 | 1 | » | - |
| #Cu3 | » | 3 | 2 | » | - |
| #Cu4 | » | 2.5 | 2.5 | » | - |
| #Cu5 | » | 2 | 3 | » | - |
| #Cu6 | » | 1.75 | 3.25 | » | - |
| #Cu-G | » | 1.75 | 3.25 | » | 3 |

### **Table S2: Summarized Raman band frequencies of CuO and Cu_2_O**

| **Compound** | **Raman Shift (cm^‑1^)** | **Raman Shift (cm^-1^) (Literature)** |
| --- | --- | --- |
| CuO | 292 | 298^1^, 298^2^ |
|  | 337 | 347^1^, 343^2^ |
|  | 608 | 597^1^, 629^2^ |
| Cu_2_O | 112-117 | 110^3^,106^4^ |
|  | 133-137 | 148^3^,146^4^ |
|  | 200-210 | 218^1^, 220^3^,216^4^ |
|  | 407-410 | 416^3^ |
|  | 516-535 | 523^1^,525^3^ |
|  | ~620 | 623^1^,635^3^,620^4^ |

### **Table S3: Summarized absorption peaks of FT-IR spectra**

| **Zone** | **Functional Group** | **Frequency (cm^-1^)** | **Frequency (cm^-1^) (Literature)** |
| --- | --- | --- | --- |
| 1 | Cu-O (CuO) | 475 | 700-500^5^, 484^6^ |
| 1 | Cu-O (CuO) | 534 | 700-500^5^, 533^7^, 530^6^ |
| 1 | Cu-O (CuO) | 586 | 700-500^5^, 580^6^ |
| 1 | Cu-O (Cu_2_O) | 620 | 700-500^5^ |
| 2 | C-H Bending | 880 | 900-700^5^ |
| 2 | C-O Stretching | 1045 | 850-1250^8^ |
| 2 | C-O Stretching | 1084 |  |
| 2 | C-O Stretching | 1123 |  |
| 2 | C-O Stretching | 1184 |  |
| 3 | C=C Stretching | 1634 | 1611.2^5^ |
| 4 | -CH_2_- | 2850 | 2845-2980^9^ |
| 4 | -CH_3_- | 2923 |  |
| 4 | -CH_3_- | 2960 |  |

### **Table S4: Summarized PEC analysis for #Cu6 C-free and #Cu6 photocathodes films under dark conditions and continuous irradiation (wavelength: 455 nm, 1333 mW**·**cm^-2^) in 0.1M NaOH.**

| **Film** | **Condition** | **Medium** | **Photocurrent Density (mA·cm^-2^) @ 0V vs. RHE** | **Onset potential**  **(V vs. RHE)** | **Overpotential (V vs. RHE)**  **@10mA/cm^2^** |
| --- | --- | --- | --- | --- | --- |
| #Cu6 C-free | Light Off | 0.1M NaOH | -0.12 | 0.179 | -0.319 |
| #Cu6 C-free | Light On | 0.1M NaOH | -0.20 | 0.188 | -0.307 |
| #Cu6 | Light Off | 0.1M NaOH | -0.11 | 0.212 | -0.320 |
| #Cu6 | Light On | 0.1M NaOH | -0.19 | 0.234 | -0.252 |

### **Table S5: Summarized component values for equivalent circuit (simulation) of #Cu6 C-free and #Cu6 in different medium**

| **Material** | **Medium** | **E_app_ vs Ag/AgCl** | **R_se_(KΩ·cm^-2^)** | **CPE-T (x10^-5^)** | **CPE-P(x10^-1^)** | **R_se_(KΩ·cm^-2^)** |
| --- | --- | --- | --- | --- | --- | --- |
| #Cu6 C-free | 0.5M Na_2_SO_4_ | -0.1V/Dark | 100±8 | 2.17±0.03 | 8.096±0.004 | 20.5±0.3 |
|  |  | -0.1V/Light | 95±8 | 3.97±0.09 | 7.073±0.006 | 9.3±0.2 |
|  |  | -0.2V/Dark | 102±8 | 1.88±0.02 | 8.366±0.005 | 38.4±0.8 |
|  |  | -0.2V/Light | 98±8 | 3.03±0.05 | 7.569±0.005 | 28.2±0.7 |
|  |  | 0V/Dark/Semic.1 | 105±8 | 7.49±0.03 | 6.754±0.001 | 1.80±0.05 |
|  |  | 0V/Dark/Semic.2 | 105±8 | 9.67±0.05 | 5.520±0.002 | 5.19±0.03 |
|  |  | 0V/Light/Semic.1 | 104±8 | 1.49±0.09 | 5.647±0.001 | 1.31±0.005 |
|  |  | 0V/Light/Semic.2 | 104±8 | 9.74±0.09 | 0.439±0.05 | 4.6±0.8 |
| #Cu6 | 0.5M Na_2_SO_4_ | -0.1V/Dark | 100±8 | 4.43±0.09 | 7.108±0.006 | 12.2±0.3 |
|  |  | -0.1V/Light | 95±8 | 6.1±0.1 | 6.457±0.006 | 4.53±0.09 |
|  |  | -0.2V/Dark | 102±8 | 3.42±0.1 | 7.690±0.005 | 38.6±0.8 |
|  |  | -0.2V/Light | 98±8 | 4.50±0.06 | 7.170±0.003 | 19.8±0.5 |
|  |  | 0V/Dark/Semic.1 | 105±8 | 7.46±0.02 | 6.64±0.02 | 2.09±0.05 |
|  |  | 0V/Dark/Semic.2 | 105±8 | 4.0±0.2 | 4.78±0.03 | 4.92±0.03 |
|  |  | 0V/Light/Semic.1 | 104±8 | 9.5±0.2 | 6.46±0.01 | 1.14±0.03 |
|  |  | 0V/Light/Semic.2 | 104±8 | 7.7±0.1 | 3.74±0.05 | 3.9±0.6 |
| #Cu6 C-free | 0.1M NaOH | -0.2V/Light | 1.12±0.01 | 2.63±0.02 | 7.737±0.010 | 13.9±0.7 |
| #Cu6 | 0.1M NaOH | -0.2V/Light | 2.15±0.01 | 1.19±0.02 | 8.896±0.003 | 16.3±0.2 |

## **Supplementary Figures**

### **Supplementary Figure 1**


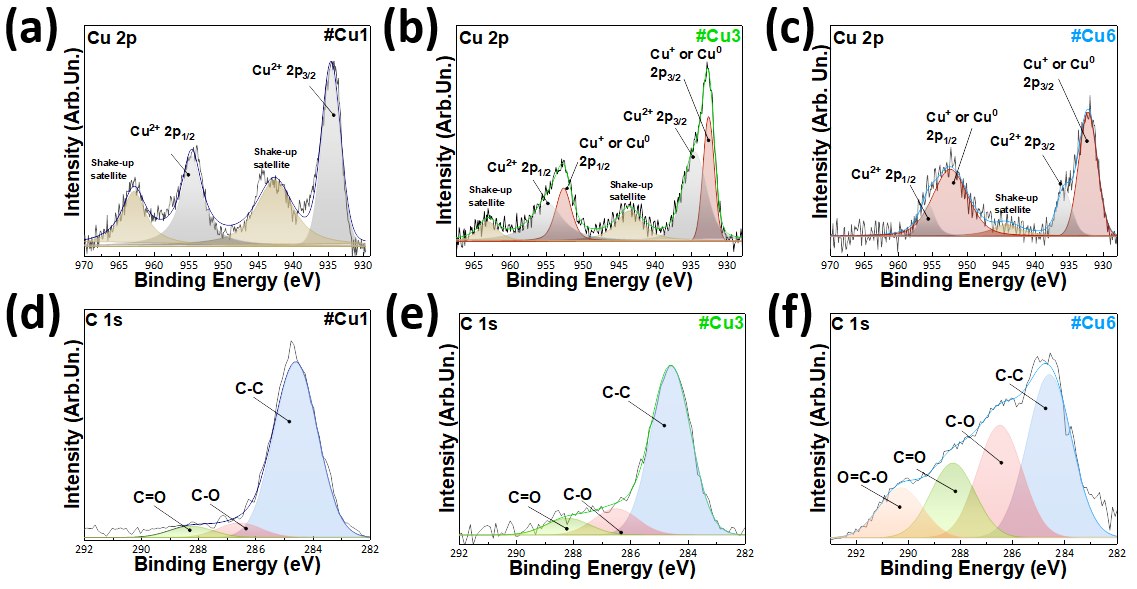


**Figure S1**: XPS analysis **a)-b)-c)** Cu 2p, **d)-e)-f)** C 1s for the materials #Cu1, #Cu3 and #Cu6

### **Supplementary Figure 2**


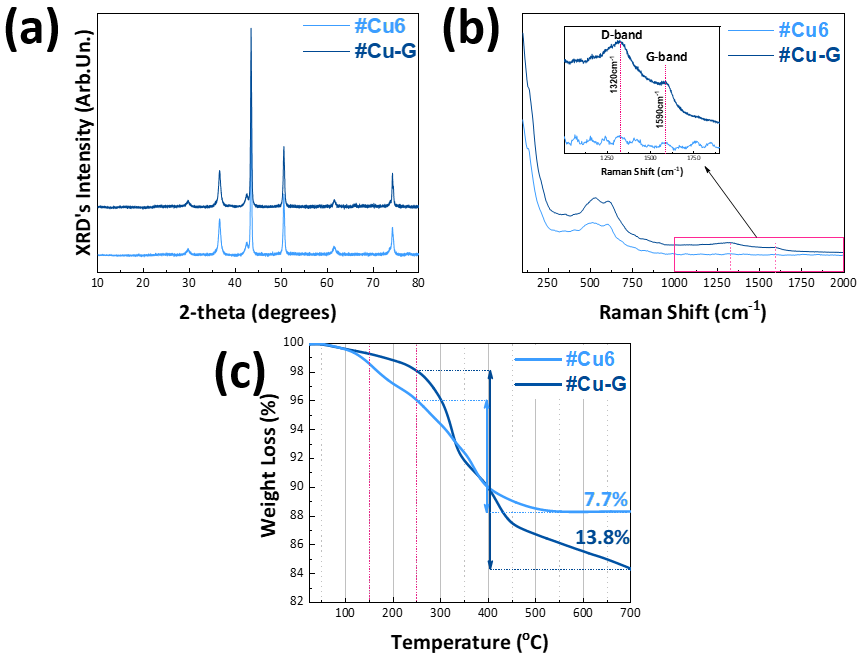


**Figure S2**: **a)** XRD patterns of #Cu6 and #Cu-G where we observe similar phase composition. **b)** Raman spectra of #Cu6 and #Cu-G. In the inset figure we can see the carbon region of these materials where we have clear evidence of carbon on the material #Cu-G whereas material #Cu6 does not exhibit the characteristic D and B-bands at 1320^‑1^ and 1590cm^‑1^ respectively. **c)** TG-DTA data of #Cu6 and #Cu-G.

### **Supplementary Figure 3**


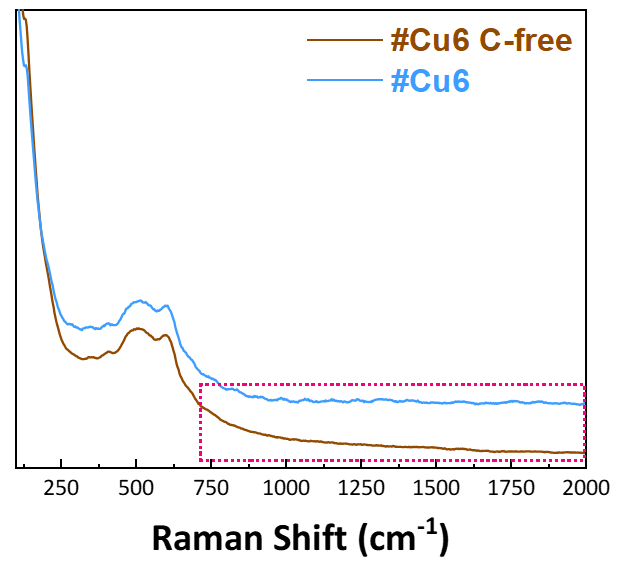


**Figure S3**: Raman spectra of #Cu6 and #Cu6 C-free materials.

### **Supplementary Figure 4**


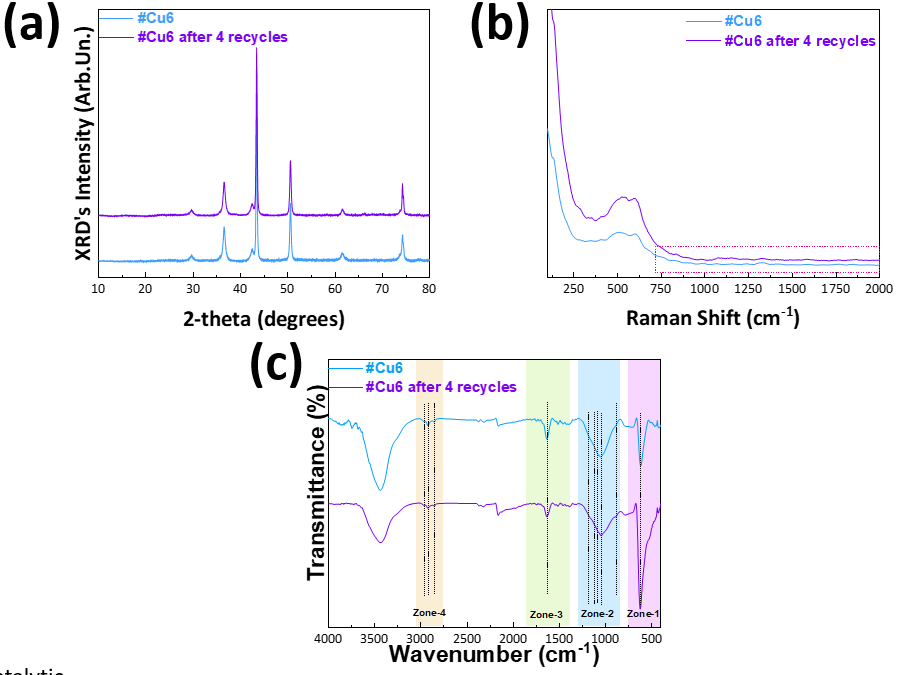


**Figure S4**: **a)** XRD patterns of #Cu6 and #Cu6 after 4 catalytic cycles where we observe similar phase composition. **b)** Raman spectra of #Cu6 and #Cu6 after 4 catalytic cycles. In the area between 750-2000cm^-1^ we observe the characteristic S-band which corresponds to non-graphitized carbon sp^2^-sp^3^. **c)** FT-IR data of #Cu6 and #Cu6 after 4 catalytic cycles.

### **Supplementary Figure 5**


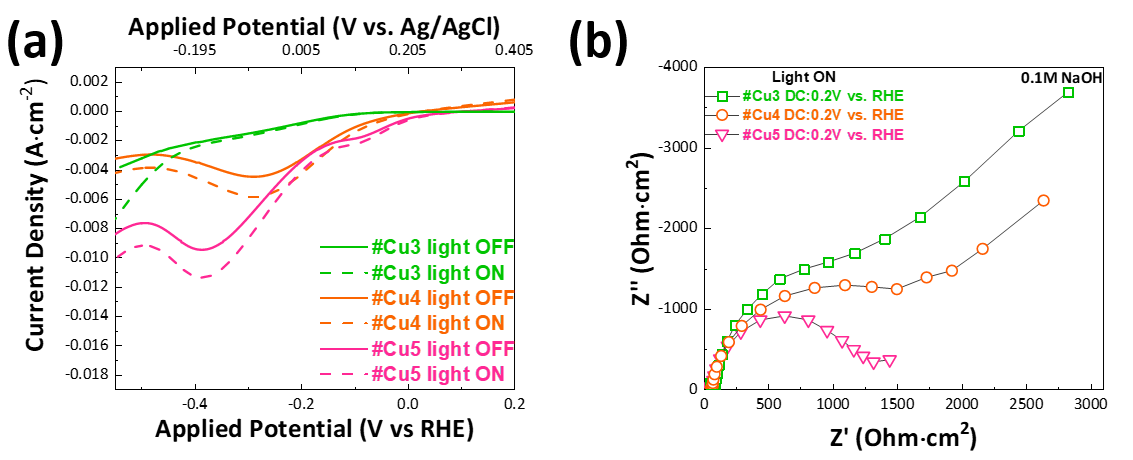


**Figure S5**: **a)** [Current Density] *vs.* [cathodic potential] data for materials #Cu3 (green line), #Cu4 (orange line) and #Cu5 (pink line), under dark (solid lines), and under continuous irradiation (dashed lines). **b)** Nyquist plot of the #Cu3 (green square symbol), #Cu4 (orange circle symbol) and #Cu5 (pink triangle symbol) under continuous irradiation.

### **Supplementary Figure 6**


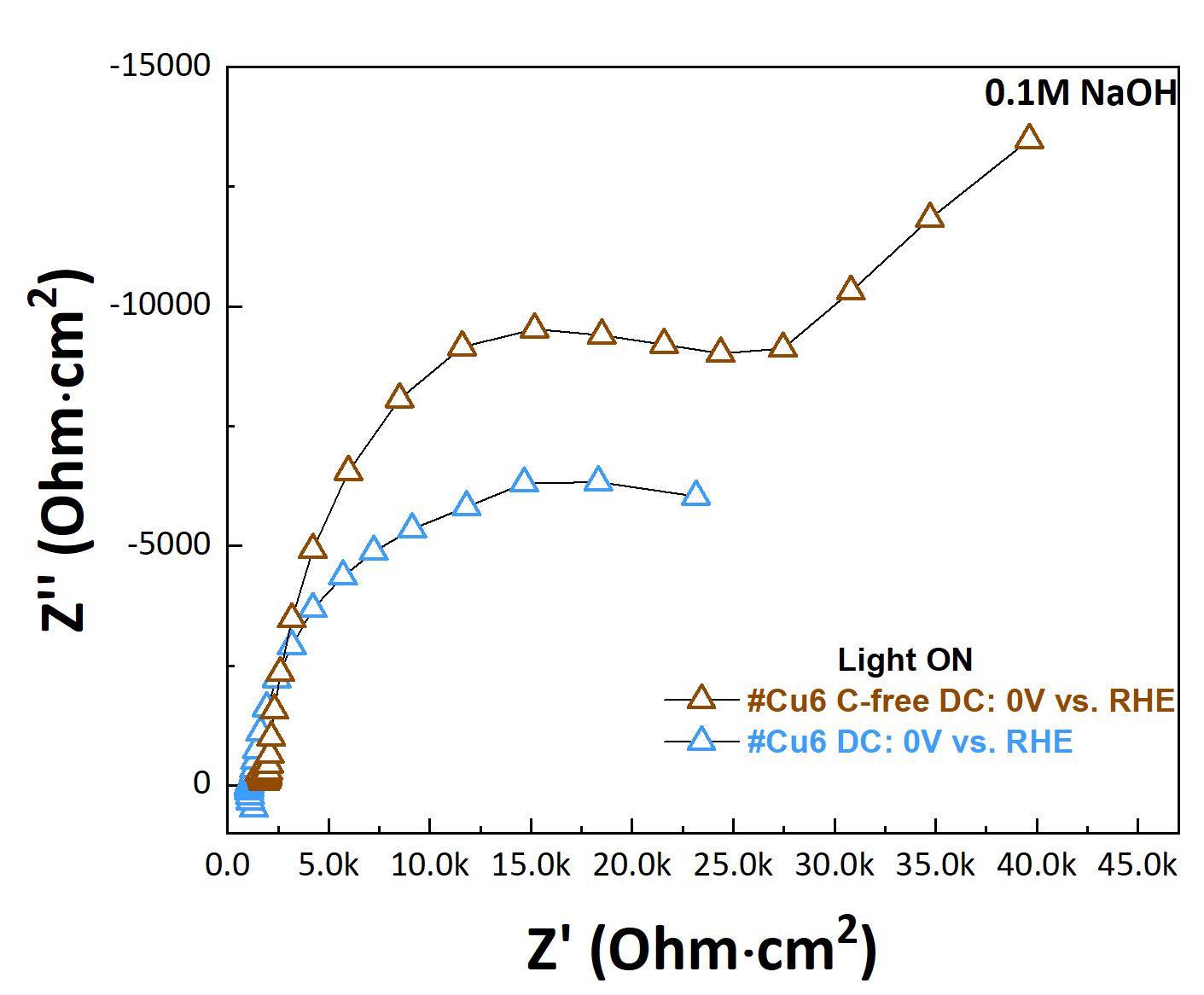


**Figure S6:** Nyquist plot of the #Cu6 C-free film (brown color) and #Cu6 film (blue color) photocathodes under continuous irradiation.

### **Supplementary Figure 7**


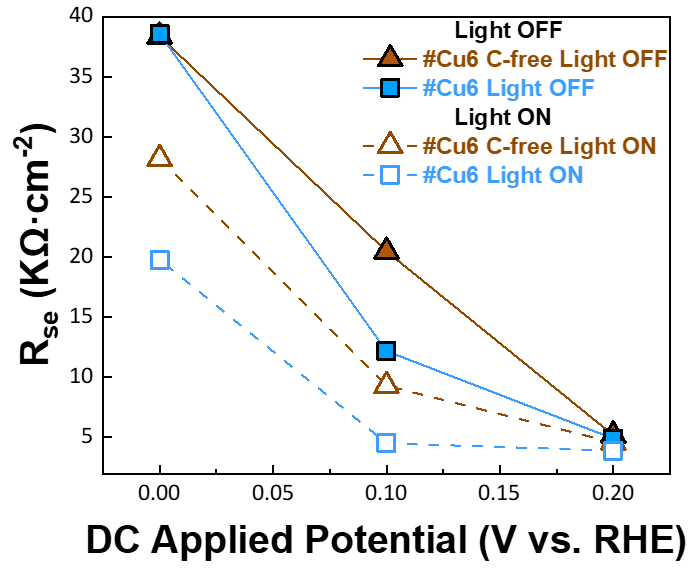


**Figure S7:** Calculation of different R_se_ values (KΩ·cm^-2^) versus different applied DC potential.

## **Apparent Quantum Yield (AQY%) Calculation**

Apparent Quantum Yield (AQY)%, was calculated using the following equations^10,11^:

$AQY=\frac{2\times N_{H2}}{2N}\times100 \%$ (1)

where N_H2_ the number of H_2_ molecules and N the incident photons (N):

$N=\frac{E\lambda}{hc}=\frac{ISt\lambda}{hc}$ (2)

where I= Light intensity (53.8 mW cm^-2^), S = Irradiation area (5 cm^2^), t= Irradiation time (h), λ = 440 nm, h = Planck constant, c =speed of light.

Making the assumption that all incident photons, $\sim2.025\times{10}^{22}$ s^-1^, were absorbed by the catalyst particles, the AQY% for the best catalyst (Cu#6 =12 μmol/h for λ=440 nm), was AQY_λ=440 nm_=0.55 %.

## **Supplementary References**

1. Deng, Y., Handoko, A. D., Du, Y., Xi, S. & Yeo, B. S. *In Situ* Raman Spectroscopy of Copper and Copper Oxide Surfaces during Electrochemical Oxygen Evolution Reaction: Identification of Cu ^III^ Oxides as Catalytically Active Species. *ACS Catal.* **6**, 2473–2481 (2016).

2. Tran, T. H. & Nguyen, V. T. Phase transition of Cu_2_O to CuO nanocrystals by selective laser heating. *Materials Science in Semiconductor Processing* **46**, 6–9 (2016).

3. Pagare, P. K. & Torane, A. P. Band gap varied cuprous oxide (Cu_2_O) thin films as a tool for glucose sensing. *Microchim Acta* **183**, 2983–2989 (2016).

4. Dhonge, B. P., Ray, S. S. & Mwakikunga, B. Electronic to protonic conduction switching in Cu_2_O nanostructured porous films: the effect of humidity exposure. *RSC Adv.* **7**, 21703–21712 (2017).

5. Sudha, V., Murugadoss, G. & Thangamuthu, R. Structural and morphological tuning of Cu-based metal oxide nanoparticles by a facile chemical method and highly electrochemical sensing of sulphite. *Sci Rep* **11**, 3413 (2021).

6. B. Tanvir, N., Yurchenko, O., Wilbertz, C. & Urban, G. Investigation of CO_2_ reaction with copper oxide nanoparticles for room temperature gas sensing. *Journal of Materials Chemistry A* **4**, 5294–5302 (2016).

7. Karthik, K., Victor Jaya, N., Kanagaraj, M. & Arumugam, S. Temperature-dependent magnetic anomalies of CuO nanoparticles. *Solid State Communications* **151**, 564–568 (2011).

8. Cintrón, M. S. & Hinchliffe, D. J. FT-IR Examination of the Development of Secondary Cell Wall in Cotton Fibers. *Fibers* **3**, 30–40 (2015).

9. Balachandran, M. Role of Infrared Spectroscopy in Coal Analysis—An Investigation. *American Journal of Analytical Chemistry* **2014**, (2014).

10. Acar, C., Dincer, I. & Naterer, G. F. Review of photocatalytic water-splitting methods for sustainable hydrogen production. *International Journal of Energy Research* **40**, 1449–1473 (2016).

11. Wang, P. *et al.* Cubic Cuprous Oxide-Based Nanocomposites for Photocatalytic Hydrogen Generation. *ACS Appl. Nano Mater.* **2**, 7409–7420 (2019).
